# Time series analysis of bovine venereal diseases in La Pampa, Argentina

**DOI:** 10.1371/journal.pone.0201739

**Published:** 2018-08-06

**Authors:** Leonardo L. Molina, Elena Angón, Antón García, Ricardo H. Moralejo, Javier Caballero-Villalobos, José Perea

**Affiliations:** 1 School of Veterinary Medicine, National University of La Pampa, General Pico, Argentina; 2 National Service of Health and Agro-Food Quality (SENASA), Centro Regional La Pampa-San Luis, Corrientes, Santa Rosa, Argentina; 3 Department of Animal Production, School of Veterinary, University of Cordoba, Córdoba, Spain; 4 Ministerio de la Producción, Gobierno de La Pampa, Santa Rosa, Argentina; ISI Foundation, ITALY

## Abstract

The venereal diseases bovine trichomoniasis (BT) and bovine genital campylobacteriosis (BGC) cause economic losses in endemic areas like La Pampa province in Argentina where beef cattle are usually extensively managed. This study used data compiled between 2007 and 2014 by a Provincial Program for the Control and Eradication of venereal diseases in order to develop and analyze retrospective models of time series for BT and BGC. Seasonality and long-term trend were explored with decomposition and simple regression methods. Autoregressive Integrated Moving Average models (ARIMA) were used to fit univariate models for the prevalence and persistence of BT and BGC. Autoregressive Integrated Moving Average with Explanatory Variable models (ARIMAX) were used to analyze the association between different time series, replacement entries and herd samplings. The prevalence and persistence of BT and BGC have decreased from 2007 to 2014. All the BT and BGC time series are seasonal and their long-term trend is decreasing. Seasonality of BT and BGC is similar, with higher rates of detection in autumn-winter than is spring-summer. Prevalence and persistence time series are correlated, indicating their changes are synchronic and follow a similar time pattern. Prevalence of BT and BGC showed the best fitting with the ARIMA (0,0,1)(0,1,1)_12_ model. While for persistence of BT and BGC, the best adjustment was with the same model with no seasonal difference where the current number of cases depends on the moving averages of the month and the previous season. Including covariates improve the fitting of univariate models, in addition, estimations using ARIMAX models are more precise than using ARIMA models. The time distribution of the samplings could be increasing the false negative ratio. According to the obtained results, the ARIMA and ARIMAX models can be considered an option to predict the BT and BGC prevalence and persistence in La Pampa (Argentina).

## Introduction

Bovine trichomoniasis (BT) and bovine genital campylobacteriosis (BGC) are two venereal diseases caused respectively by *Tritrichomonas foetus* protozoan and the Gram (-) *Campylobacter fetus* subspecies *venerealis* [1, 2]. Both agents are transmitted during coitus and colonize the reproductive tract of cows and bulls. In cows the infection can cause reproductive failure, including repeat of estrus, early embryonic death and abortions; while in bulls both infections are typically asymptomatic [3, 4]. Both infected cows and bulls can become persistent asymptomatic carriers [5].

BT and BGC affect endemically in zones where cattle production is more extensive based on communal pastures and natural breeding, where it is difficult to control the factors that typically favor these diseases [6]. Moreover, there is neither effective treatment nor vaccination, which makes even more difficult to implement control plans in the endemic zones [7]. Consequently, both diseases are still associated to economic losses due to breeding failure.

In Argentina, BT and BGC are considered endemic and are among the main causes of low reproductive efficiency [8, 9, 10]. The province of La Pampa includes around 6% of the national herd [11], and beef cattle production accounts for almost 16% of the province’s gross domestic product [12]. Awareness of the economic importance of this sector and the concern about the low reproductive efficiency led to the implementation of a Provincial Program for the Control and Eradication (PCE) of BT and BGC in 2006. Engagement in PCE is mandatory for all herds, and positive animals are culled within 120 days.

In the last decades, time series methodologies have been included in the epidemiological analysis of infectious diseases. The key feature of time series is that observations are not independent of each other, and thus future estimates are explained by previous data, and not by independent variables. The advantages and the conditions for applicability are specific for each model of analysis, and they also depend on the data that constitute the time series [13]. Decomposition methods have been used to analyze seasonality and long-term trend of venereal diseases [14]. Autoregressive integrated moving average models (ARIMA) are widely applied in infection time series modelling [15]. These are dynamic models where estimations are based on lineal combinations of previous data and its residuals. ARIMA models have been helpful to identify and predict dispersion patterns of infectious diseases in different regions, such as influenza in USA, dengue in Brazil or malaria in Bhutan [16, 17, 18]. Time series can also be used to study the association among several variables that change over time and influence each other. This is the case of autoregressive integrated moving average with explanatory variable models (ARIMAX), which allow the inclusion of series of covariate factors to the ARIMA model. This method has made it possible to explore the influence of covariation factors in the behavior of some diseases. For instance, the relationship between climate variables and leptospirosis transmission in Thailand or the human transmission of bovine brucellosis in South Korea [19, 20].

Data compiled under the PCE provide an opportunity to investigate the time pattern of BT and BGC, as well as the association with risk factors over time. This information should be important to optimize the performance of PCE and to identify appropriate measures to reduce BT and BGC occurrence. Thus, the aim of this study was to analyze the uni- and multi- variate time series of BT and BGC in La Pampa from 2007 to 2014. Decomposition methods are used to explore the seasonality and long-term trend of BT and BGC. ARIMA models are used to model the univariate time series, and ARIMAX models to explore the association among different time series.

## Material and methods

### Study area and population

The study area was the province of La Pampa in Argentina, which included around 6% of the total cattle population of Argentina [11]. La Pampa is located in the geographical center of Argentina and covers an area of 143,440 km^2^; approximately 5.2% of Argentina. Cattle production in La Pampa is typically extensive and involves two main production systems: herds that produce calves for fattening establishments (breeding herds), and herds where breeding, rearing and fattening are carried out on the same premises (full-cycle herds).

**Fig 1:**
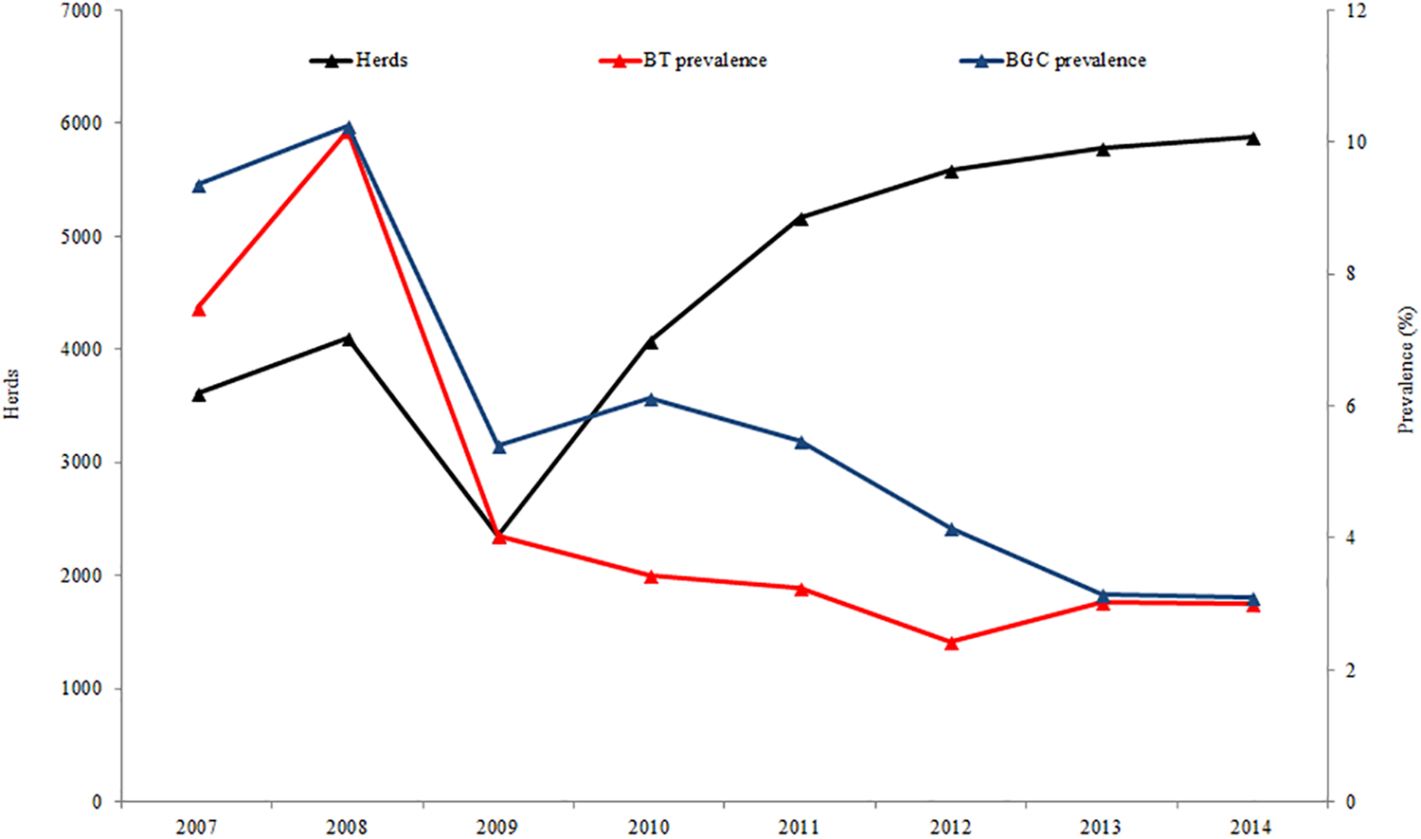
Herds and annual prevalence of BT and BGC during 2007–2014 in La Pampa (Argentina).

The study population consist of all herds tested (from 2,000 to 6,000) under the Control and Eradication Program (PCE) from 2007 January 1^st^ to 2014 December 31^st^ (Fig 1). La Pampa regulation requires the testing of all non-virgin bulls existing in a herd for BT and BGC prior to interprovincial or intraprovincial movement of any type of cattle (breeding bulls, non-breeding bulls, cows, calves) to another herd, feedlot or slaughterhouses [12]. Therefore, the study population corresponds to all herds existing in La Pampa between 2007 and 2014, except those few herds without animal movements during this period.

All non-virgin bulls in La Pampa are tested twice a year as part of PCE. The methodology of sampling and diagnosis has been thoroughly described by Molina et al. [21]. A bull was classified as negative if all results in two consecutive tests were negative, and positive if at least one test yielded positive results [22]. Herds with at least one positive bull were classified as positive.

### Data

The present study used the data compiled and reported by PCE, and added on a monthly basis from 1^st^ January 2007 to 31^st^ December 2014. Prevalence and persistence of BT and BGC was analyzed. Prevalence is defined as the proportion of positive herds over the total tested herds. Persistence is defined as the proportion of positive herds in year *n* that were also positive in year *n– 1* over the total tested herds. The following variables were evaluated as possible risk factors, also added monthly: tested herds, tested herds persistent to BT, tested herds persistent to BGC, entry of breeding bulls for replacement, entry of breeding cows for replacement, and entry of heifers for replacement.

### Decomposition methods

Decomposition methods are used to obtain seasonality and long-term trend that characterize the behavior of the time series:

Timeseries=Seasonality+Trend+Residual (1)

Seasonality of the time series is expressed using seasonal indices, calculated as the monthly average of the ratio between the monthly value and the annual monthly average value [23]. The seasonal index varies between 0 and 1 when the monthly value is lower than the medium level, and it is higher than 1 in the opposite case. Spearman’s rank correlation coefficients were calculated in order to analyze the seasonal relationship between time series.

The seasonality of time series is removed dividing each value of the series by its corresponding seasonal index, obtaining the non-seasonal time series. The relationship between the non-seasonal time series (dependent variable) and time (independent variable) was modeled using simple linear least square regression in order to obtain the long-term trend [14]. Different alternative models were built and evaluated. The fitting of the model was determined by the coefficient of determination, (R^2^) and it was contrasted performing analysis of variance (ANOVA) [24].

### ARIMA model

ARIMA model is a univariate analysis procedure widely used to model the occurrence of infectious diseases. ARIMA model results from a differencing process based on autoregressive moving average model (ARMA), which expresses the actual value as a linear combination of the previous value (autoregressive component) and the residual series (moving average) [25]. ARMA model can be expressed as:

$$y(t)=\varphi_{y}(t-1)+\cdots +\varphi_{p}y(t-p)+\varepsilon (t)-\theta_{1}\epsilon (t-1)-\cdots -\theta_{q}\varepsilon (t-q)$$ (2)

where *y*(*t*) denotes the value of the series in the *time t*; *ε*(*t*) the residual through time *t*; *φ* and *θ* are its corresponding coefficients.

ARIMA model is expressed as ARIMA (p, d, q) x (P, D, Q)s, where p, d and q are non-negative integers referring to the order of the autoregressive, integrated and moving average parts of the model respectively; whereas P, D and Q represent the order of the seasonal autoregressive, differencing and moving average respectively. The subscripted letter “s” denotes the seasonal period length.

The ARIMA modeling procedure was introduced by Box and Jenkins, and consists of three iterative steps: identification, estimation and verification of diagnosis [26]. Before the identification step, data must be stationary, which can be achieved by performing an appropriate seasonal difference in addition to the regular difference of the ARIMA model. Stationarity can be tested using the method developed by Dickey-Fuller [27]. The identification step includes the process of determining seasonal and non-seasonal orders using the autocorrelation and partial autocorrelation functions of the differenced series [18, 28]. Parameters in the model are estimated using the method of conditional least squares after the identification step [29]. Finally, the adequacy of the established model for the series is verified by performing white noise test to check if the residuals are independent and normally distributed [30]. It is possible that several ARIMA models can be identified, so it is required to choose an optimum model. This optimum model is generally determined based on the Akaike information criterion (AIC) and on Schwartz Bayesian criterion (SBC) [31].

### ARIMAX model

In contrast with the ARIMA model, where the previous values of the dependent variable (AR) and residual series (MA) are taken after the differencing process, the ARIMAX model is an extension of ARIMA modeling incorporating an independent explanatory variable [20]. ARIMAX model can be expressed as follows:

$$y(t)=\beta_{x}(t)+\varphi_{y}(t-1)+\cdots +\varphi_{p}y(t-p)+\varepsilon (t)-\theta_{1}\epsilon (t-1)-\cdots -\theta_{q}\varepsilon (t-q)$$ (3)

where *x*(*t*) denotes a covariate at time *t*; *β* denotes its associate coefficient; *y*(*t* − 1)…*y*(*t* − *p*) denotes the previous values, and *ε*(*t*)…*ε*(*t* − *q*) are the residual series. For each time series, all the rest were used as covariate. Non-significant covariates (p>0.05) were removed from the model. As well as in ARIMA, several ARIMAX models can be identified. The selection of the optimum ARIMAX model is also based on AIC and SBC.

Before fitting the ARIMAX model, cross-correlations between the different analyzed time series were calculated [32]. The ARIMAX model hypothesizes that the time series *y*(*t*) is related to past lags of the *x*(*t* + *m*) series. The cross-correlations can help to identify lags of the *x*-variable that could be used as predictors of variable *y*(*t*). The value of cross-correlation is between -1 and +1. Absolute values closer to 1 indicate a higher correlation between both series.

ARIMA and ARIMAX models were fitted to the series of prevalence and persistence of BT and BGC from 2007 to 2013 and tested by estimating prevalence and persistence for 2014. All statistical analyses were performed with a signification level of alpha ≤ 0.05 using the software SPSS version 15.0.

## Results

Seasonal indices were obtained from the original time series and reveal the seasonality of the testing of herds, replacement entries, BT and BGC prevalence and persistence (Table 1). Correlations between the seasonal indices are shown in Table 2.

**Table 1:** Seasonal indices of different time series analyzed during 2007–2014 in La Pampa (Argentina).

Time series	Jan	Feb	Mar	Apr	May	Jun	Jul	Aug	Sep	Oct	Nov	Dec
BT prevalence	0.78	1.06	1.00	0.37	2.26	1.13	1.00	0.79	0.83	0.40	0.46	0.24
BT persistence	0.82	1.97	0.62	2.30	1.25	1.35	1.26	1.50	0.36	0.22	0.16	0.18
BGC prevalence	0.40	0.93	0.79	0.52	2.29	1.18	1.05	0.91	0.98	0.44	0.52	0.30
BGC persistence	0.55	1.06	0.52	2.43	1.57	2.22	1.48	1.38	0.34	0.25	0.20	0.00
Entry of breeding bulls	0.69	0.43	0.40	0.56	0.58	0.98	0.65	1.10	1.69	2.49	1.49	0.96
Entry of breeding cows	0.73	0.84	0.70	1.22	1.41	1.40	1.21	1.23	0.88	0.83	0.69	0.84
Entry of heifers	0.92	1.54	0.67	0.99	1.00	0.95	0.87	0.70	0.98	0.99	1.27	1.12
Persistent BT tested herds	0.24	0.11	0.47	0.35	0.90	0.71	1.10	1.92	2.74	1.69	0.97	0.79
Persistent BGC tested herds	0.15	0.13	0.42	0.48	0.92	1.05	1.22	1.82	3.08	1.61	0.73	0.83
Tested herds	0.21	0.16	0.26	0.31	0.39	0.61	0.97	1.90	2.80	2.45	1.22	0.73

**Table 2:** Spearman correlations between the seasonal indices of different time series analysed during 2007–2014 in La Pampa (Argentina).

	**BTi**^1^	**BTp**^2^	**BGCi**^3^	**BGCp**^4^	**EB**^5^	**EC**^6^	**EH**^7^	**pBT**^8^	**pBGC**^9^	**TH**^10^
BTi	1									
BTp	ns	1								
BGCi	0.95	ns	1							
BGCp	ns	0.86	ns	1						
EB	ns	-0.60	ns	ns	1					
EC	ns	0.60	0.66	0.85	ns	1				
EH	ns	ns	ns	ns	ns	ns	1			
pBT	ns	ns	ns	ns	0.70	ns	ns	1		
pBGC	ns	ns	ns	na	0.63	ns	ns	0.98	1	
TH	ns	ns	ns	ns	0.86	Ns	ns	0.95	0.91	1

^1^BTi: BT prevalence,

^2^BTp: BT persistence,

^3^BGCi: BGC prevalence,

^4^BGCp: BGC persistence,

^5^EB: entry of breeding bulls,

^6^EC: entry of breeding cows,

^7^EH: entry of heifers,

^8^pBT: persistent BT tested herds,

^9^pBGC: persistent BGC tested herds,

^10^TH: tested herds.

The seasonality of the testing of the herds and the testing of BT and BGC persistent herds is considerably high, and it is concentrated from August to October, with a seasonal peak in September. The three-time series follow a similar seasonal pattern with correlation coefficients over 0.90.

The seasonality of the entry of breeding bulls for replacement is also quite pronounced and it is concentrated from August to November, with a seasonal peak in October. Bulls entered tend to coincide with the testing of the herds, with correlation coefficients between 0.60 and 0.86. The entry of breeding cows for replacement is less seasonal than for breeding bulls and is concentrated from April to August, while the entry of heifers for replacement is slightly seasonal.

The prevalence of BT and BGC follows a similar seasonal pattern, with a correlation coefficient of 0.95. Both diseases have a higher prevalence in autumn to winter (May-July) than in spring to summer (October-December), with a seasonal peak in May. No more significant correlations were identified.

Persistent cases of BT and BGC are more frequent in autumn—winter than in spring—summer, with a seasonal peak in April. Both series follow a similar seasonal pattern, with a correlation coefficient of 0.86. Persistence of BT and BGC tends to coincide with the entry of breeding cows for replacement, while the entry of breeding bulls for replacement shows a negative seasonal trend with the persistence of BT.

The long-term trend for each disease is obtained through simple regression models for the time series removing seasonality. The model, the estimation of the coefficient and the adjusted coefficient of determination (R^2^), are shown in Table 3. The best fitted model was BGC persistence (R^2^ = 79.56), while BT persistence model did not show good fitting (R^2^ = 28.83). Prevalence models showed an acceptable and intermediate adjustment. The trends of persistence and prevalence of BT and BGC are decreasing. Prevalence of BGC decreases slower than prevalence of BT, while persistence of BGC decreases faster than persistence of BT.

**Table 3:** Regression model for each time series removed seasonality during 2007–2014 in La Pampa (Argentina).

Time series	Model	*β*_0_	*β*_1_	R^2^	P
BT prevalence	$Y={\left(\beta_{0}+\beta_{1}\sqrt{X}\right)}^{2}$	4.3652	-2.4969	68.85	0.0000
BT persistence	$Y=\beta_{0}+\beta_{1}\sqrt{X}$	2.3614	-2.0322	28.83	0.0000
BGC prevalence	$Y={\left(\beta_{0}+\beta_{1}\sqrt{X}\right)}^{2}$	4.1164	-1.7524	62.10	0.0000
BGC persistence	$Y=\sqrt{a+\beta_{1}/X}$	-1.4527	1.0247	79.56	0.0000

ARIMA models were established for the four-time series from 2007 to 2013. Models were tested by estimating values for the year 2014. The orders of the autoregressive process and moving average were determined from the autocorrelation and partial autocorrelation functions [25]. Several ARIMA models were adjusted. Results from the estimations are displayed in Table 4.

**Table 4:** ARIMA models with the best adjustments for time series of prevalence and persistence of BT and BGC from 2007–2013 in La Pampa (Argentina).

Time series	Model	AIC^1^	SBC^2^
BT prevalence	**ARIMA (0,0,1)(0,1,1)**_**12**_	**2.180**	**2.494**
	ARIMA (1,0,1)(0,1,1)_12_	2.181	2.497
	ARIMA (1,0,0)(0,1,1)_12_	2.263	2.494
	ARIMA (0,0,1)(1,1,1)_12_	2.271	2.556
	ARIMA (1,0,0)(1,1,1)_12_	2.279	2.556
BT persistence	**ARIMA (0,0,1)(0,0,1)**_**12**_	**-1.115**	**-1.704**
	ARIMA (1,0,0)(0,0,1)_12_	-1.087	-1.695
	ARIMA (1,0,1)(0,0,1)_12_	-0.994	-1.573
	ARIMA (1,0,0)(1,0,1)_12_	-1.085	-1.621
	ARIMA (0,0,1)(1,0,1)_12_	-0.991	-1.604
BGC prevalence	**ARIMA (0,0,1)(0,1,1)**_**12**_	**1.859**	**2.342**
	ARIMA (1,0,0)(0,1,1)_12_	1.876	2.342
	ARIMA (0,0,1)(1,1,1)_12_	1.900	2.413
	ARIMA (1,0,0)(1,1,1)_12_	1.953	2.413
	ARIMA (1,0,0)(1,1,0)_12_	1.934	2.370
BGC persistence	**ARIMA (0,0,1)(0,0,1)**_**12**_	**-0.420**	**-1.469**
	ARIMA (1,0,0)(0,0,1)_12_	-0.406	-1.298
	ARIMA (0,0,1)(1,0,1)_12_	-0.408	-1.090
	ARIMA (0,0,1)(1,0,0)_12_	-0.419	-1.469
	ARIMA (1,0,0)(1,0,0)_12_	-0.409	-1.298

^1^AIC: Akaike information criterion,

^2^SBC: Schwartz Bayesian criterion

The ARIMA model finally selected for each series is highlighted and corresponds to that which minimizes the Akaike Information Criterion (AIC) and the Schwartz Bayesian criterion (SBC). Prevalence of BT and BGC present the same ARIMA pattern. The model that better adjusted both series is ARIMA (0,0,1)x(0,1,1)_12_. This implies that after a seasonal differentiation, time *t* prevalence is relevant for the residual at times *t*, *t-1*, t-*12* and *t-13*. Persistence of BT and BGC also present the same ARIMA pattern, although with no seasonal differentiation.

Cross correlations between different time series were calculated. The highest correlation coefficients and lags are shown in Table 5. The highest values appear when lags are 0 except for the entrance of breeding bulls, which shows a strong positive correlation with testing of herds series in lag -1. Testing of herds and testing of persistent BT and BGC herds show high and positive correlations with each other. The prevalences of BT and BGC are correlated, and the persistence and prevalence of each disease are also correlated, although poorly.

**Table 5:** The highest cross-correlation coefficients between the different time series analyzed during 2007–2013 in La Palma, Argentina (the values in the brackets are the lags).

	**BTi**^1^	**BTp**^2^	**BGCi**^3^	**BGCp**^4^	**EB**^5^	**EC**^6^	**EH**^7^	**pBT**^8^	**pBGC**^9^	**TH**^10^
BTi	1									
BTp	0.64 (0)	1								
BGCi	0.68 (0)	0.46 (0)	1							
BGCp	0.58 (-1)	0.37 (-1)	0.47 (0)	1						
EB	-0.23 (0)	0.24 (5)	Ns	Ns	1					
EC	ns	0.24 (1)	Ns	Ns	0.83 (-4)	1				
EH	ns	ns	Ns	0.28 (9)	0.47 (-3)	0.53 (-6)	1			
pBT	0.27 (6)	ns	0.25 (6)	0.27 (4)	0.80 (-1)	0.69 (3)	-0.40 (2)	1		
pBGC	ns	0.25 (4)	Ns	0.25 (4)	0.84 (-1)	0.64 (3)	0.46 (7)	0.82 (0)	1	
TH	-0.27 (-11)	-0.30 (-2)	-0.26 (-11)	0.26 (-1)	0.82 (-1)	0.72 (3)	-0.39 (2)	0.72 (0)	0.83 (0)	1

^1^BTi: BT prevalence,

^2^BTp: BT persistence,

^3^BGCi: BGC prevalence,

^4^BGCp: BGC persistence,

^5^EB: entry of breeding bulls,

^6^EC: entry of breeding cows,

^7^EH: entry of heifers,

^8^pBT: persistent BT tested herds,

^9^pBGC: persistent BGC tested herds,

^10^TH: tested herds.

**Fig 2:**
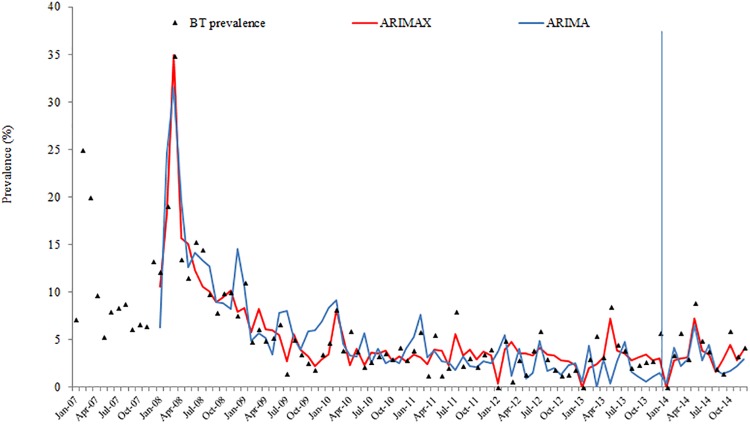
BT prevalence fitting and testing performance by ARIMA and ARIMAX (the vertical blue line separates modelling from estimates).

**Fig 3:**
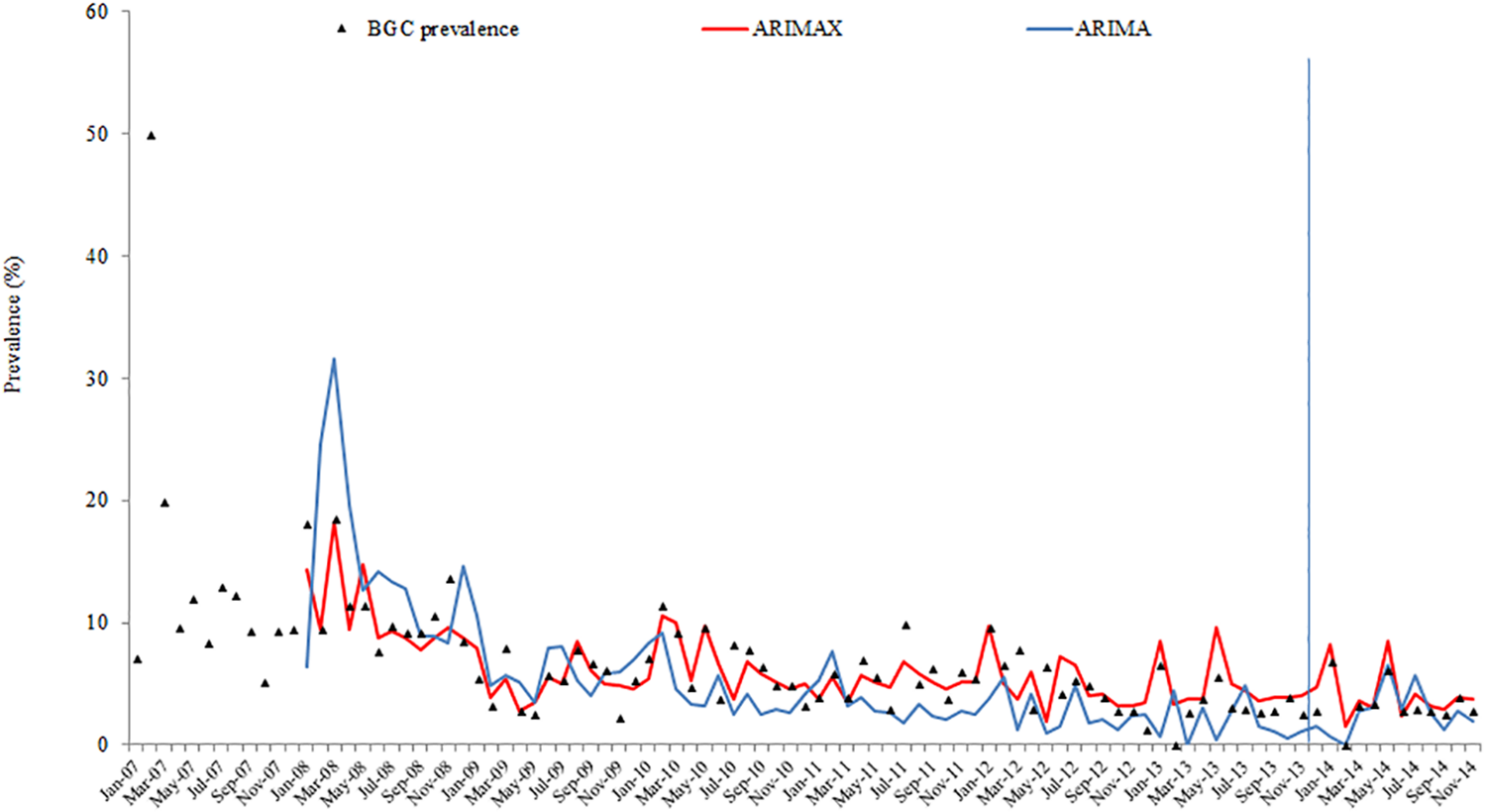
BGC prevalence fitting and testing performance by ARIMA and ARIMAX (the vertical blue line separates modelling from estimates).

**Fig 4:**
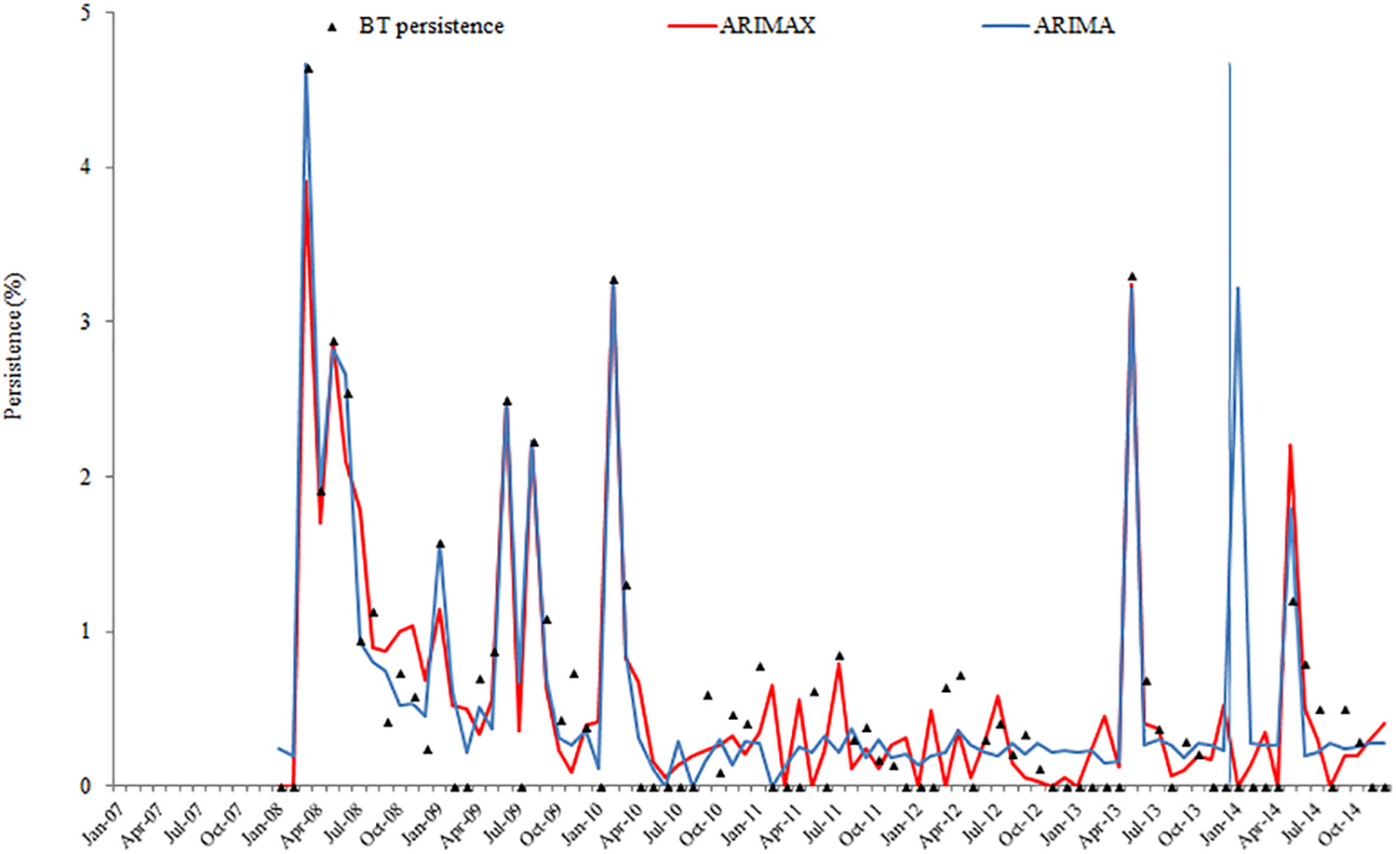
BT persistence fitting and testing performance by ARIMA and ARIMAX (the vertical blue line separates modelling from estimates).

**Fig 5:**
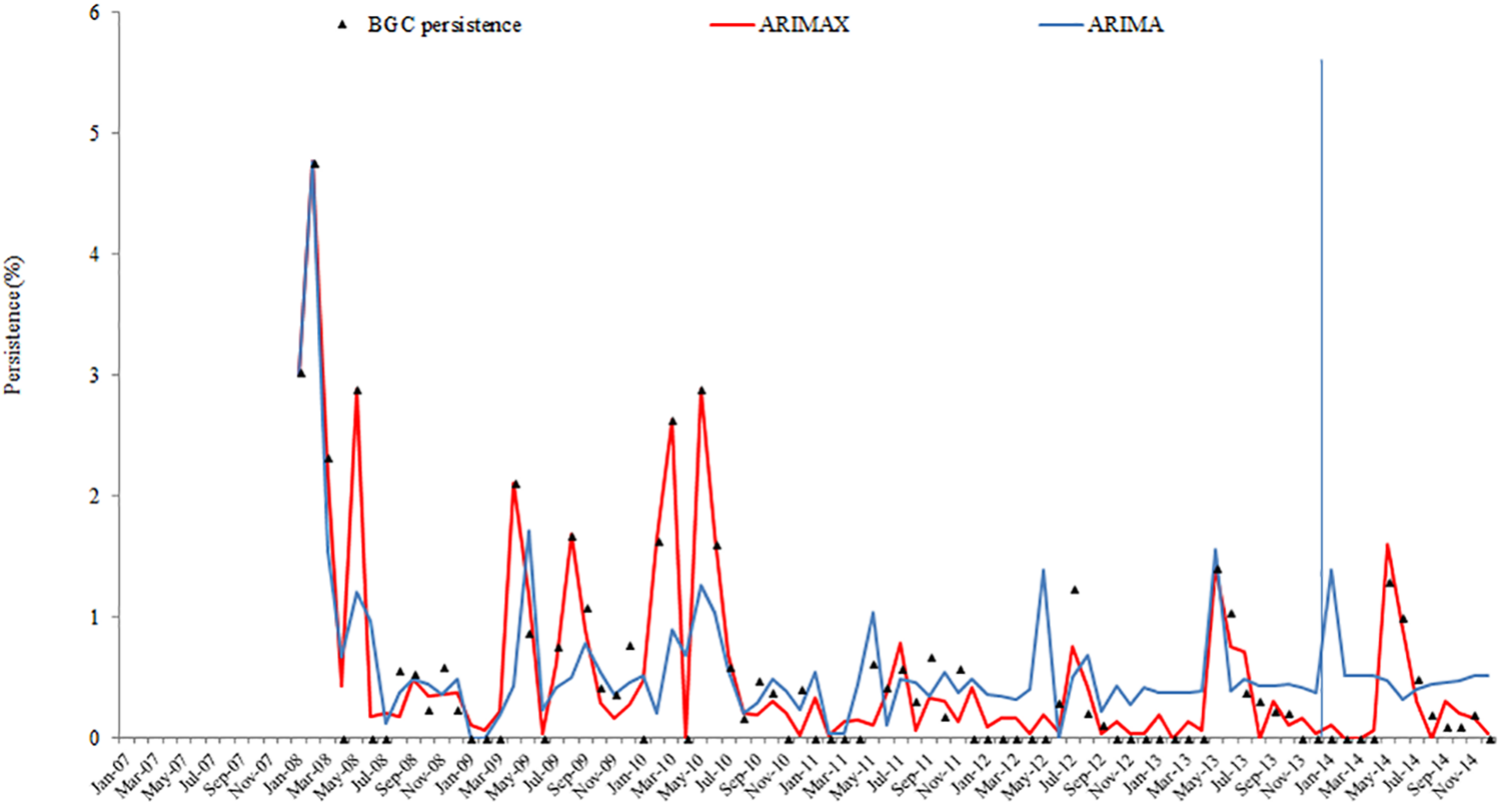
BGC persistence fitting and testing performance by ARIMA and ARIMAX (the vertical blue line separates modelling from estimates).

Results from ARIMAX models for the four-time series are presented in Table 6. The best ARIMAX model is highlighted in Table 6 according to the AIC and SBC criteria. ARIMAX models obtained smaller AIC and SBC than their corresponding univariate ARIMA models, which indicates that the fitting performance improved with the inclusion of the covariate variables. The fitting and prediction of prevalence and persistence of BT and BGC for the ARIMA and ARIMAX models are plotted in Figs 2, 3, 4 and 5.

**Table 6:** ARIMAX models with the best adjustments for time series of prevalence and persistence of BT and BGC during 2007–2013 in La Pampa (Argentina).

Time series	Model	Covariates	AIC^1^	SBC^2^
BT prevalence	ARIMAX (1,0,0)(0,0,0)_12_	BGC prevalence	1.321	1.714
	ARIMAX (1,0,1)(0,0,0)_12_	BGC prevalence	1.342	1.713
	ARIMAX (0,0,0)(0,0,1)_12_	BGC prevalence	1.346	1.769
	ARIMAX (0,0,0)(1,0,0)_12_	BT persistence	1.213	1.648
	ARIMAX (1,0,0)(1,0,0)_12_	BT persistence	1.345	1.628
	ARIMAX (0,0,0)(1,0,0)_12_	BT persistence	1.432	1.648
	ARIMAX (1,0,0)(0,0,1)_12_	BGC prevalence, BT persistence	1.456	1.621
	**ARIMAX (0,0,0)(1,0,0)**_**12**_	**BGC prevalence, BT persistence**	**1.123**	**1.582**
	ARIMAX (1,0,0)(1,0,1)_12_	BGC prevalence, BT persistence	1.345	1.588
BT persistence	ARIMAX (1,0,0)(0,0,0)_12_	BT prevalence	-1.134	-1.311
	**ARIMAX (0,0,1)(0,0,0)**_**12**_	**BT prevalence**	**-1.252**	**-1.337**
	ARIMAX (1,0,1)(0,0,0)_12_	BT prevalence	-1.211	-1.330
BGC prevalence	ARIMAX (1,0,1)(1,0,0)_12_	BT prevalence	1.456	1.886
	ARIMAX (1,0,0)(0,0,0)_12_	BT prevalence	1.753	1.873
	ARIMAX (1,0,1)(0,0,0)_12_	BT prevalence	1.564	1.843
	ARIMAX (0,0,0)(1,0,0)_12_	BGC persistence	1.542	1.842
	ARIMAX (1,0,1)(0,0,0)_12_	BGC persistence	1.675	1.985
	ARIMAX (0,0,0)(1,0,1)_12_	BGC persistence	1.764	1.906
	ARIMAX (0,0,1)(1,0,0)_12_	BT prevalence, BGC persistence	1.565	1.825
	**ARIMAX (0,0,1)(0,0,0)**_**12**_	**BT prevalence, BGC persistence**	**1.310**	**1.750**
	ARIMAX (0,0,1)(0,0,1)_12_	BT prevalence, BGC persistence	1.968	1.825
BGC persistence	**ARIMAX (0,0,1)(1,0,0)**_**12**_	**BGC prevalence**	**-1.975**	**-1.821**
	ARIMAX (0,0,1)(0,0,0)_12_	BGC prevalence	-1.834	-1.808
	ARIMAX (0,0,1)(0,0,1)_12_	BGC prevalence	-1.763	-1.823

^1^AIC: Akaike information criterion,

^2^SBC: Schwartz Bayesian criterion

Root mean squared error (RMSE) between the observed value of the raw series and the estimated values obtained through the ARIMA and ARIMAX model were compared (Table 7). All RMSE for ARIMAX model were lower than the ARIMA model in the modeling process and point estimation.

**Table 7:** Comparison of the performances of the ARIMA and ARIMAX models.

Time series	RMSE modelling		RMSE estimates	
	ARIMA	ARIMAX	ARIMA	ARIMAX
BT prevalence	2.827	1.639	2.743	1.554
BGC prevalence	2.620	1.949	2.312	1.817
BT persistence	0.381	0.290	0.256	0.145
BGC persistence	0.326	0.250	0.234	0.265

## Discussion

Data collected in La Pampa (Argentina) over 8 years was used to assess the viability of developing time series models using monthly changes in BT and BGC in the province. The rates of prevalence of BT and BGC peaked in March 2008 and February 2007, respectively. Since then they have decreased, demonstrating effective control measures. The effort of PCE to control both diseases has also been able to decrease persistent cases of BT and BGC. The initial prevalence of both diseases was generally lower than those reported in other endemic areas in Asia, Australia, North America, South America and South Africa [4, 9, 33, 34, 35, 36, 37, 38, 39, 40, 41, 42, 43]. A prevalence of 37% for BGC was reported in Uruguay, while no herds were found to be positive to BT [44]. In Buenos Aires (Argentina) a prevalence of 1.5% was reported for BGC and of 19.4% for BT [45]. In the east of La Pampa, a prevalence of 11.1% was found for BT and of 7.0% for BGC [46]. Most of the reported prevalences come from studies conducted with few herds and limited conditions, so it is possible that they do not reflect accurately the situation [7]. For instance, in South Africa, the occurrence of BGC seems to be vastly underestimated [6].

ARIMA models were used to fit univariate time series of prevalence and persistence of BT and BGC. Models have proved to be appropriate to fit the historic prevalence and persistence, and to estimate prevalence and persistence in 2014. All-time series followed the same pattern, where the present number of cases depends mainly on the moving averages of the previous month and season. ARIMAX models were also fitted to explore correlation between time series of both diseases and potential co-variation factors. The inclusion of covariates improved the adjustment of univariate models. In addition, estimations in ARIMAX models are more accurate than in ARIMA models. ARIMAX models add predictive value in the control of both diseases. Thus, prevalence not only can be estimated using historical data, but also using persistence of the same disease and prevalence of the other one.

Adjusted models have properly captured the trend of prevalence and persistence of BT and BGC, although they predict future occurrence with medium precision. The relatively high RMSEs are due to the small number of cases, in both absolute and relative terms compared to the population monthly tested [20, 47]. On the other hand, factors related to herd management were not considered in the models, which can play an important role in exploring the time series relationship among both diseases [36, 48]. These data are not currently available, although they could be included in future studies.

ARIMA and ARIMAX models have been typically used in econometrics. However, their use is increasing in the medical field [16, 17, 18]. Zhang et al. [14] used them to model the background and future incidence of syphilis in China, evidencing a higher performance when different types of syphilis were included as covariates. Soebiyanto et al. [17] modelled the transmission of influenza and obtained a high predictive capacity including climatic factors. Wu et al. [49] modeled the occurrence of dengue in Taiwan and also obtained better results when including climate variables as covariates.

The main advantage of these models is that they consider seasonal differences, which can be useful to predict sexually transmitted diseases [14]. However, their implementation requires appropriate datasets, as those compiled by epidemiological surveillance systems, which are currently scarce for livestock venereal diseases [7, 15]. This could explain that, although several papers that apply time series models in veterinary epidemiology have been recently published (i.e. sylvatic rabies, Hussain et al. [50]; bovine fasciolosis, Silva et al. [51]; Newcastle disease, Mubamba et al. [52]), we have not found any studies analyzing time series of livestock venereal diseases.

Cross correlation between time series of both diseases is high when lag is zero, what indicates that changes are synchronic. Correlations between seasonal indices also indicate that BT and BGC follow a similar seasonal pattern that is generally defined by a higher frequency in autumn—winter than in spring—summer. These results suggest that BT and BGC are bound to similar temporal factors. However, the evaluated co-variation factors related to testing of herds and the entrance of replacement cattle have not been significant predictors in any ARIMAX model. In addition, no strong or significant correlations have been evidenced with any of the different time series of both diseases.

There are no evident biological or transmission explanations for a lag of 0 between persistence and prevalence of the same disease. It is probably explained by the contribution of the persistent cases to the monthly prevalence, particularly relevant in the early years of the study. This means that we use the same time persistence to estimate the prevalences, potentially decreasing the predictive benefit. In ARIMAX, the covariates can be a burden of modeling if the cross-correlations are not high enough [14]. This is a limitation of the method.

Seasonality of BT and BGC could be related to the reproductive cycle of herds. Although in most of herds the reproduction is handled with a continuous season, mating tends to be concentrated in spring—summer, due to a better forage offer that stimulates the beginning of the estrous cycle. On the other hand, in autumn—winter bulls are usually at reproductive rest. Szonyi et al. [53] found in Texas (USA) a higher prevalence of BT in summer than in winter, which they related to a higher bad quality bull testing frequency in summer. Reproductive inactivity increases the concentration of microorganisms in the preputial fluid and, therefore, facilitates detection of both diseases [21, 54]. Consequentially, detection rates fluctuate seasonally with the reproductive cycle, being higher during periods of inactivity.

The reproductive cycle also constitutes a temporary factor that should be considered when planning the testing of herds. In La Pampa, the testing of the herds should be concentrated in autumn—winter in order to reduce the risk of false negatives generated by low concentration of microorganisms in the preputial fluid [21, 54]. However, herds are generally tested in spring, when bulls are generally active. Thus, there is a high risk of false negatives generated by this factor. Consequently, the prevalence of both diseases should be higher than reported by PCE; probably close to the prevalence obtained during autumn—winter months.

Another factor that should be taken into account is the entrance of breeding bulls for replacement into herds. Herds should be tested after the entrance of the bulls, even if they were certified as free of venereal diseases, since the risk of false negative due to a low concentration of microorganisms is high. However, the entrance of bulls and the testing of herds occur with a lag of one month. This suggests that tests tend to take place a month before the entrance of breeding bulls into the herds; with no effective knowledge of their sanitary status. In agreement with Ondrak [55], this factor is relevant to explain the spread of both diseases in free herds.

The identified temporal and seasonal patterns should be considered together with risk and handling factors in order to improve the PCE success. Compiled data from PCE previously allowed the identification of spatial patterns of BT and BGC in the province, revealing spatial correlation between the risk of BT and BGC [21]. Other studies have shown that BT and BGC share some of their main risk factors [9, 10, 36, 56, 57]. Thus, the development of integrated actions focused on the common features of BT and BGC should improve effectiveness and efficiency of the intervention measures [57].

The identified long-term trends suggest that persistent cases of both diseases are drastically reduced, while the prevalence of BT and BGC has been decreased to reach minimum levels, difficult to reduce further considering the actual conditions of PCE. These results are similar to those achieved by other control plans based on policies of “testing and culling” for bovine venereal diseases. For instance, in Wyoming (USA), a control plan similar to PCE managed to reduce the herd-level prevalence of BT to 1.29% in nine years [58]. In the breed Asturiana de la Montaña (Spain), a voluntary control test plan using the “test and cull” approach managed to reduce herd-level prevalence of BT from 41% to 19.7% in two years [59]. These results suggest that, in agreement with Yao [54] and Collantes-Fernandez et al. [59] diagnostic and slaughter schemes are effective in reducing the occurrence rates. However, the complete elimination of BT and BGC without substantial changes in management appears unlikely, because putative risk factors associated with both diseases are present in the management of farms.

Currently, the sampling schedule is a result of individual decisions of each farmer, who establishes in which moment the herd is sampled. This way of planning is the optimum when the farmer applies productive criteria, since from a sanitary perspective the best moment for test the herd coincides with the best moment from a zootechnical point of view. From a sanitary perspective, the testing of herds should take place during the non-reproductive season, and at least a month after the entrance of breeding cattle into the herd [53, 54, 55]. This sampling system minimizes the probability of obtaining false negatives and the transmission of both pathogens to free herds. From a zootechnical point of view, this system allows the detection of both diseases before they can cause reproductive failures, which also allows the replacement with free bulls without generating substantial delays in the reproductive cycle.

However, most farmers do not take this approach. Sampling schedule probably responds to the need of testing the herd before the authorization of cattle movement, which in La Pampa is compulsory for any destination, even to slaughterhouse. An important reason for this could be that farmers underestimate the scale of the problem and its impact on the farm economy. In fact, Jiménez et al. [10] showed that the status of BGC is not a sensitive issue for Argentinean farmers. There are currently no global governmental monitoring programs to track the incidence and prevalence of bovine venereal diseases [7]. In general, there are no regulations requiring testing for bulls sold by private treaty or those resident in privately owned herds [55]. Both diseases are typically asymptomatic or subclinical, so it is difficult to take notice of their severity until the occurrence of reproductive failures. In addition, practices such as keeping a reproductive activity record, daily inspection of the herd, pregnancy diagnosis or culling of non-pregnancy cows are not commonly applied, which reduces the possibility of identifying precociously reproductive problems [9, 10]. Jointly, all these factors make the global livestock industry underestimate the adverse effects of bovine venereal diseases.

Persistent herds have been dramatically reduced, which can be explained by an increased awareness of the severity of the problem after suffering the disease. Rae et al. [39] reported that farmers familiarized with BT diminished the risk of disease. In Spain, infection by *T*. *foetus* has been recently found to increase calving intervals by 79 days and reduce income by 68.7% in herds, while implementation of a voluntary testing and culling policy was effective in improving reproductive efficiency [59]. More than 50% of reproductive failure in Argentina has been estimated to be caused by venereal diseases, although the arising productive and economic consequences have never been formally assessed [60]. In this sense, additional research could be useful to understand the economic and productive consequences of both diseases in the region, as well as to provide evidence of the effectiveness of the intervention measures. On the other hand, the managing authority could plan the sampling jointly with the farmers, at least temporarily, in order to correct the deviations identified in the present study.

In addition to the sampling schedule other factors should be considered to improve the rates of detection and to reduce the risk of false negatives. On one side, in La Pampa two consecutive negative tests are enough to declare a bull as disease-free, while the recommendation is to obtain three or four negatives [22]. On the other side, there are available diagnostic techniques based on PCR that significantly improve the results obtained by the official ones implemented by PCE, and only requiring one test *per* animal [61, 62].

## Conclusions

Time series analysis was an effective tool for modeling the historical and future prevalence of *T*. *foetus* and *C*. *fetus* infections in La Pampa (Argentina). ARIMAX models showed superior performance than ARIMA models for the modeling of BT and BGC prevalences. Results showed that both diseases are seasonal and synchronous, with detection rates in autumn-winter than in spring-summer. Our results also provide valuable data for planning control strategies and making recommendations to farmers and managing authorities. Testing and culling policy was effective in reducing prevalence and persistence of *T*. *foetus* and *C*. *fetus*. Knowing the status of the bulls before breeding is the first step for developing a sound control plan and the best form of herd surveillance. However, the complete elimination of BT and BGC without substantial changes in management appears unlikely, because putative risk factors associated with both venereal diseases are present in the management of these farms.
